# Unravelling the Significance of Cystatin C and Bunina Bodies in Amyotrophic Lateral Sclerosis Pathogenesis

**DOI:** 10.1111/nan.70083

**Published:** 2026-06-29

**Authors:** Sarah M. Granger, Rosemary A. Staniforth, Asbjorg Osk Snorradottir, Johnathan Cooper‐Knock, Kurt J. De Vos, J. Robin Highley

**Affiliations:** ^1^ University of Sheffield Sheffield UK; ^2^ Department of Pathology Landspitali—The National University Hospital of Iceland Reykjavik Iceland; ^3^ Arctic Therapeutics Reykjavik Iceland; ^4^ Faculty of Medicine University of Iceland Reykjavik Iceland

**Keywords:** amyotrophic lateral sclerosis, Bunina bodies, cystatin C, motor neuron disease

## Abstract

Amyotrophic lateral sclerosis (ALS), also known as motor neuron disease (MND), is a fatal neurodegenerative disease primarily affecting motor neurons. Two key protein inclusions found in lower motor neurons serve as neuropathological hallmarks of the disease in human tissue: the TDP43‐positive inclusion and the cystatin C‐positive Bunina body. Despite their diagnostic specificity and presence in most sporadic and familial ALS cases, Bunina bodies remain poorly understood, and their true prevalence is likely underestimated. The co‐occurrence of the Bunina body and the TDP43 inclusion may provide valuable insights into the development of TDP43 pathology in ALS. Thorough characterisation of the Bunina body is needed to understand this interplay and the broader pathomechanisms of disease. This review examines our current knowledge of Bunina bodies and the biochemical properties of cystatin C that may promote its aggregation. Sequestration and aggregation of cystatin C into Bunina bodies may diminish its neuroprotective functions, including cysteine protease inhibition, autophagy induction and anti‐amyloidogenic activity, thereby contributing to ALS pathogenesis. This review also evaluates findings from human post‐mortem tissue and ALS disease models, discussing the value and limitations of these models in the context of Bunina bodies and TDP43 pathology. Finally, we discuss cystatin C's use as a biomarker and its therapeutic potential. A deeper understanding of cystatin C biology, its relationship with TDP43 pathology and improved ALS models will be essential for determining whether targeting cystatin C could provide a viable avenue for future ALS therapies.

## Summary

- Cystatin C‐positive Bunina bodies are a disease‐specific hallmark of ALS, but their prevalence has likely been underestimated.
- In ALS, TDP43 pathology is observed significantly more frequently in lower motor neurons that also contain Bunina bodies, suggesting a mechanistic link.
- Cystatin C has varied neuroprotective functions that may be lost in ALS when Bunina bodies are formed.
- Targeting cystatin C—to stabilise its soluble form or prevent its aggregation into Bunina bodies—may offer therapeutic potential, but this first requires better disease models and a deeper mechanistic understanding.

**Abbreviations**
ALS: amyotrophic lateral sclerosis
ALSFRS‐R: ALS Functional Rating Scale–Revised
APP: amyloid precursor protein
C9orf72: chromosome 9 open reading frame 72
CNS: central nervous system
CSF: cerebrospinal fluid
fALS: familial amyotrophic lateral sclerosis
FTD: frontotemporal dementia
FTLD: frontotemporal lobar degeneration
FUS: fused in sarcoma
HCCAA‐I: Icelandic hereditary cystatin C amyloid angiopathy
H&E: haematoxylin and eosin
MND: motor neuron disease
NAC: N‐acetyl‐cysteine
sALS: sporadic amyotrophic lateral sclerosis
SOD1: superoxide dismutase 1
TARDBP: TAR DNA‐binding protein 43
TDP43: transactive response DNA‐binding protein of 43 kDa

## 1 Introduction

Amyotrophic lateral sclerosis (ALS) is the most common form of motor neuron disease (MND), describing a group of neurodegenerative disorders affecting motor neurons. In Europe, the incidence of ALS ranges from 1.11 to 5.55 per 100,000 person‐years, and the prevalence ranges from 3.44 to 10.80 per 100,000 population [1]. Significant variability in both incidence and prevalence occurs between geographical regions [2], which have differing genetic landscapes from European populations [3]. This low prevalence but relatively high risk is due to the disease's rapid and untreatable nature. Upper and lower motor neurons degenerate, leading to muscle weakness and spasticity, progressing to respiratory failure and death approximately 3–5 years after diagnosis. In the United Kingdom, the terms ‘MND’ and ‘ALS’ are often used synonymously because ALS is the most common clinical manifestation of MND. Therefore, ‘ALS’ will be used throughout this review.

Like many neurodegenerative diseases, ALS has a combination of both genetic and sporadic origins. Approximately 10% of ALS cases are familial (fALS), with mutations being described in 50+ genes that mostly follow an autosomal dominant inheritance pattern [4]. However, a substantial number of apparently ‘sporadic’ ALS patients are mutation carriers in large clinic and population datasets, suggesting that reduced penetrance is a general feature of ALS‐determining genes [5, 6]. The most common mutations are in chromosome 9 open reading frame 72 (*C9ORF72*), superoxide dismutase 1 (*SOD1*), TAR DNA‐binding protein (*TARDBP*) and fused in sarcoma (*FUS*) [7]. However, 90%–95% of patients develop sporadic ALS (sALS) and have considerable clinical and pathological overlap with fALS. Currently, there is no cure for ALS, and disease‐modifying treatments are limited. The US Food and Drug Administration has only three approved drugs. Riluzole and edavarone both have only modest effects on lifespan. Of these, only riluzole is approved within Europe [8]. The third, tofersen, is an antisense oligonucleotide targeting the *SOD1* gene to reduce toxic protein levels. It has been recently approved in both the United States and Europe and is only available to the subset of patients with *SOD1* mutations [9].

ALS is the clinical diagnosis for MND with both upper and lower motor neuron signs and is named after the key pathological correlates of these: amyotrophy (muscle wasting) and lateral sclerosis (degeneration of the corticospinal tracts—the axons of upper motor neurons), respectively. However, there are many other neuropathological changes. In the brain, upper motor neurons and the intervening neuropil degenerate in the motor cortex, leading to atrophy of the precentral gyrus [10]. Degeneration also occurs in specific motor brainstem nuclei, most notably the facial and hypoglossal nuclei, and there can be degeneration of the medullary pyramids. Prefrontal and temporal cortex atrophy can be observed in patients who develop dementia and is most pronounced in those individuals who receive a clinical diagnosis of frontotemporal dementia (FTD, corresponding to a pathological diagnosis of frontotemporal lobar degeneration). As some forms of FTD have genetic and pathological overlap with ALS, it is considered to be on the same disease spectrum [11]. In the spinal cord, lower motor neurons in the anterior horns degenerate, and there is thinning of the ventral nerve rootlets and white matter degeneration, particularly within the corticospinal tracts [10, 12]. Motor neuron degeneration is not the only cellular feature of ALS. There is a marked microglial reaction [13], and an increased GFAP+ reactive astrocyte phenotype can be observed in post‐mortem tissue in comparison to neurologically healthy controls (reviewed by Ashford et al. [14]). Oligodendrocytes harbour protein aggregates, and reduced myelin basic protein and demyelination are features of ALS [15].

**FIGURE 1:**
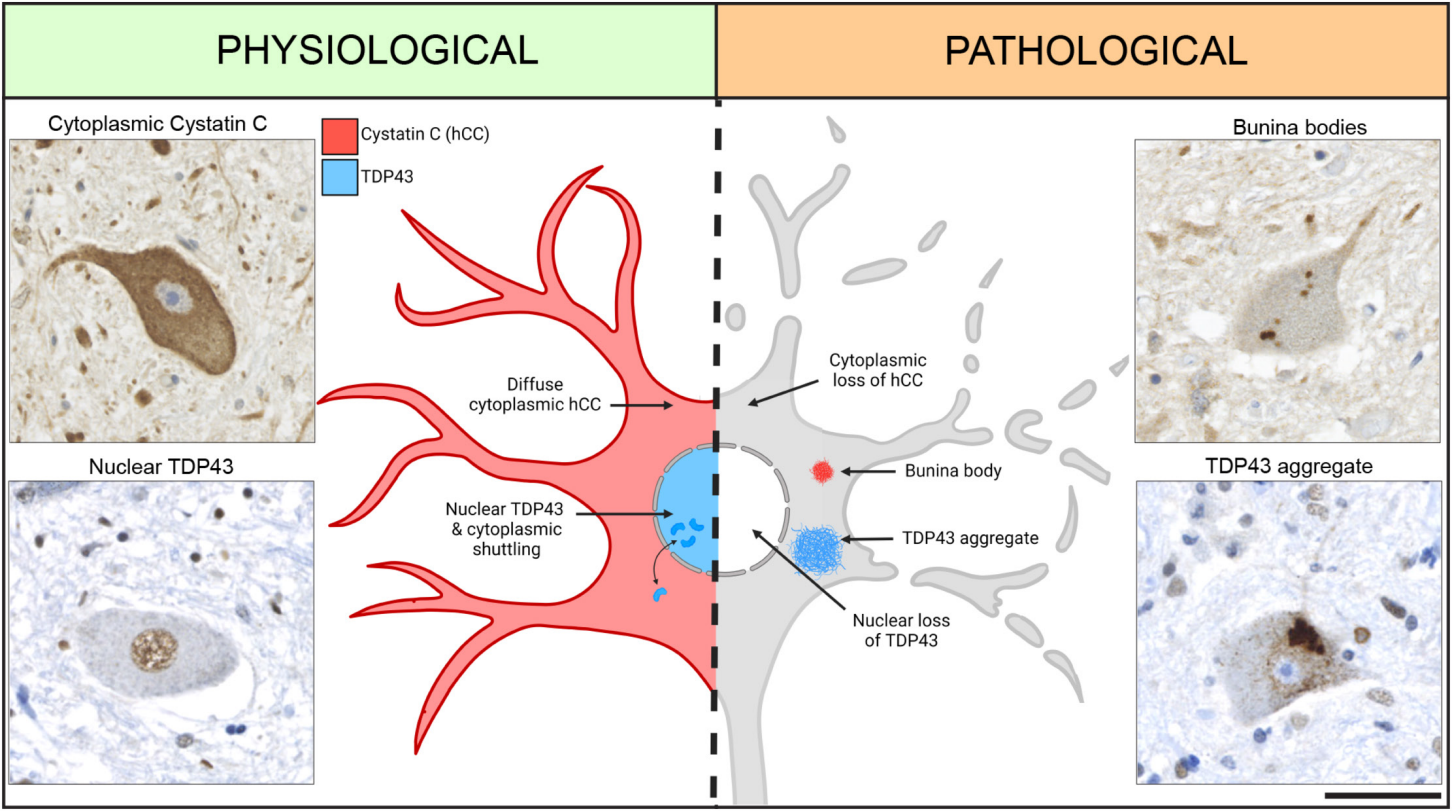
Cystatin C and TDP43 in lower motor neurons in neurologically healthy controls and ALS. Healthy/physiological lower motor neurons have high levels of cytoplasmic cystatin C and nuclear TDP43 (left). Degenerating/pathological lower motor neurons have depletion of cytoplasmic cystatin C and Bunina bodies and nuclear loss and cytoplasmic aggregation of TDP43 (right). Created with BioRender.com. For histological images, scale bar = 50 μm.

At autopsy, protein aggregates within remaining motor neurons and oligodendrocytes are pathological hallmarks of disease: Ubiquitylated cytoplasmic inclusions containing the transactive response DNA‐binding protein of 43‐kDa (TDP43) protein are observed in approximately 97% of ALS cases [16, 17]. Under physiological conditions, approximately 90% of the protein resides in the nucleus. In ALS, TDP43 vacates the nucleus and forms cytoplasmic aggregates (Figure 1). The protein is often truncated into carboxyl‐terminal fragments that can be hyperphosphorylated and ubiquitylated (reviewed by Prasad et al. [18]). Over 50 causative mutations in the *TARDBP* gene that encodes TDP43 have been identified in ALS. However, these mutations only account for approximately 4% of fALS cases, whereas TDP43 inclusions are observed in 97% of all ALS cases, including sporadic and other genetic forms [19]. TDP43 aggregation is therefore a pathological hallmark of ALS. Although TDP43 aggregates are central to most forms of ALS, they can also be seen in a number of conditions, now collectively termed TDP43 proteinopathies [20]. This raises the question of what differentiates ALS from other TDP43 proteinopathies and what causes TDP43 proteinopathy in the majority of ALS cases that do not have *TARDBP* mutations. In addition, secondary TDP43 aggregates can be seen alongside other protein aggregates in a number of other neurodegenerative conditions, such as Alzheimer's disease [21] and Parkinson's disease [22]. The precise contribution of TDP43 pathology to these conditions is the subject of debate [23]. Therefore, if TDP43 pathology can be an effect rather than a cause of other pathological processes, it may be possible that TDP43 pathology could be a secondary pathology in cases that are devoid of *TARDBP* mutations. One candidate explanation relates to the second pathological hallmark of ALS: the Bunina body.

This review will focus on the Bunina body and the significance of the key protein within it, cystatin C. Despite its universal and specific association with ALS, the Bunina body remains poorly understood. This stands in contrast to the relatively extensive work on ubiquitylated TDP43‐positive inclusions (reviewed by Prasad et al. [18]). This review will summarise the research characterising the Bunina body, possible mechanisms of cystatin C aggregation, the neuroprotective or toxic roles of cystatin C and the interplay between TDP43 and cystatin C. We conclude that the formation of the Bunina body could be a factor in driving the pathogenesis of ALS and that there is the possibility of manipulating cystatin C for future therapeutic interventions.

## 2 The Bunina Body

The Bunina body is a pathological inclusion that is specific to ALS and has not been observed in any other diseases to date [24]. The inclusion, observed in lower motor neurons, was first described in two cases of fALS in 1962 by Tat'yana Bunina, a neuropathologist from the USSR [25]. They were described as a possible neurotrophic virus, but later investigations failed to support transmissibility or viral origins. The origins of Bunina bodies are still unknown.

**FIGURE 2:**
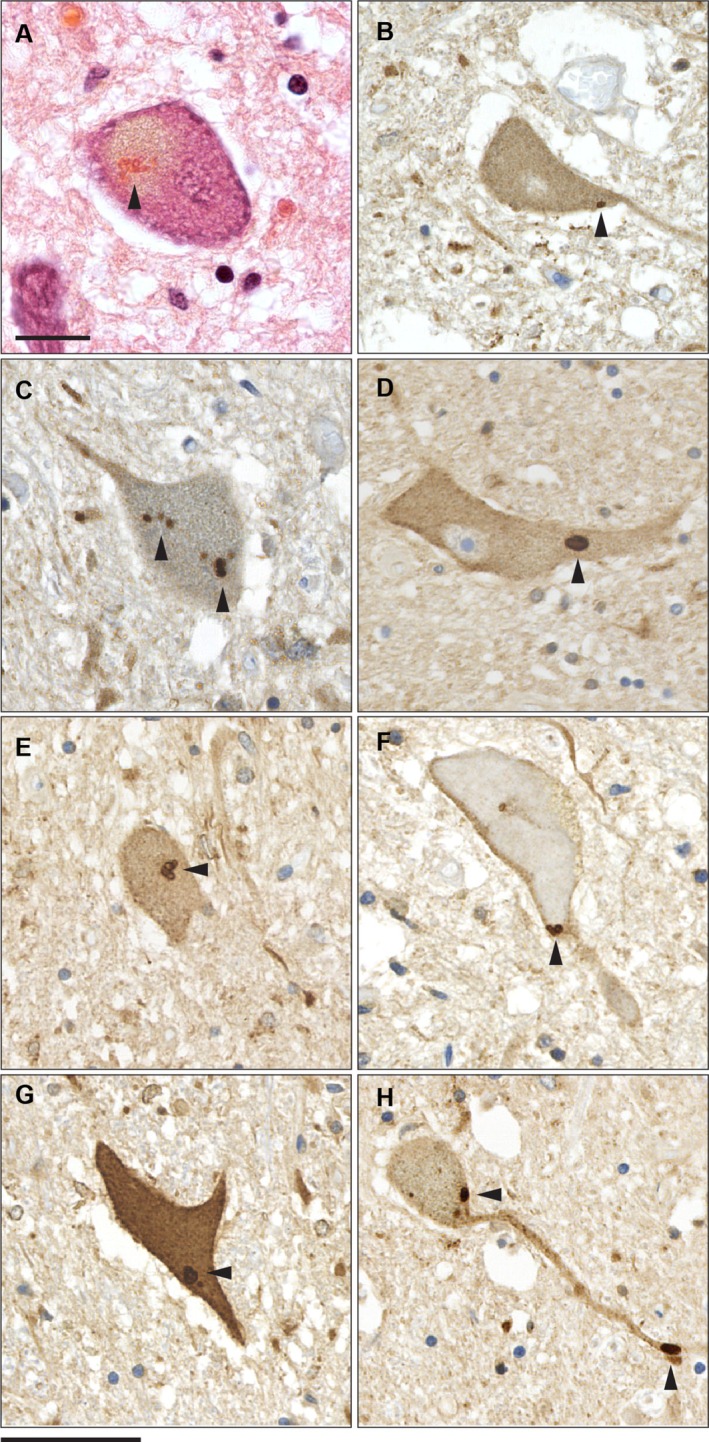
Bunina bodies in ALS post‐mortem tissue. (A) Bunina bodies appear as intensely eosinophilic inclusions via H&E staining. Via anti‐cystatin C immunohistochemistry, their morphology is diverse, appearing as single small (B) or large (D) inclusions, or multiple inclusions throughout the cytoplasm (C). They can be clustered and have immuno‐negative central regions (E), and appear rarely in both chromatolytic (F) and cystatin C‐positive (G) neurons. Bunina bodies are also observed in neuronal processes (H). Scale bar (A) = 20 μm. Scale bar (B–H) = 50 μm.

These intracytoplasmic inclusions are small (1–5 μm), round or oval‐shaped and are intensely eosinophilic (Figure 2). Electron microscopy shows Bunina bodies to contain an amorphous electron‐dense material and have no limiting outer membrane, but are surrounded by vesicular or tubular structures [24]. Their morphology is diverse: they can be observed as single, rounded inclusions, occasionally with weak immunoreactivity in central regions or in chains and clusters of multiple Bunina bodies throughout the cytoplasm or within neuronal processes. Importantly, they are immunoreactive for cystatin C [26] and are most commonly seen in neurons with reduced cystatin C immunoreactivity [27]. They can very rarely be observed in a neuron with high levels of cytoplasmic cystatin C and in neurons undergoing chromatolysis (Figure 2).

Bunina bodies are most frequently observed in lower motor neurons in ALS [24, 27, 28, 29, 30]. They are infrequently reported in Betz cells [31], the locus coeruleus [32], medullary reticular formation [33] and other regions [34]. Bunina bodies in non‐ or extra‐motor regions appear rare but may indicate the spread of this pathology over a prolonged or particularly aggressive disease course. These findings may also contribute to the heterogeneity in the ALS population.

Bunina bodies are observed in sALS [29], *C9orf72*‐fALS [35, 36], and usually in cases with rare genetic mutations associated with TDP43 pathology such as angiogenin (*ANG*), annexin A11 (*ANXA11*), ataxin‐2 (*ATXN2*) and *DNAJC7* [37, 38, 39, 40]. In contrast, they are not associated with *SOD1*‐fALS [41], which lacks TDP43 pathology. Although *FUS*‐fALS also lacks TDP43 proteinopathy, it has been reported that Bunina bodies are present in some [42], but not all cases [43, 44] via haematoxylin and eosin (H&E) staining. The largest post‐mortem examination of sALS estimated that 88 of 102 cases (86%) demonstrate this pathology. The authors used H&E alone, on an unspecified number of sections per case, to determine this [29]. In other publications, as few as three out of 13 sALS cases (23%) had Bunina bodies, as demonstrated via immunohistochemistry on three sections per case [45]. This variability in prevalence likely comes from the chosen detection method, number of cases or number of sections analysed (see Table 1).

**TABLE 1:** Summary of the reported prevalence of Bunina bodies.

Ref.	ALS cases with Bunina bodies (%)	No. of sections	Detection method
[46]	22/23 (95.6%)	Unknown	H&E
[47]	20/31 (64.5%)	Unknown	H&E
[48]	38/43 (88.3)	Unknown	H&E
[49]	9/16 (56.3%)^a^	Unknown	H&E & IHC
[30]	19/20 (95%)^b^	Unknown	IHC
[29]	88/102 (86.3%)	Unknown	H&E
[50]	24/28 (85.7%)	Unknown	H&E & IHC
[45]	3/13 (23.1%)	Three sections	IHC
[27]	6/9 (66.7%)	Three sections	IHC
[51]	15/18 (83.3%)	Five serial sections	H&E
[28]	16/20 (80%)	Unknown	H&E
[52]	18/20 (90%)	Unknown	H&E & IHC
[53]	8/12 (66.7%)	Unknown	H&E

Abbreviations: H&E, Haematoxylin & Eosin; IHC, anti‐cystatin C immunohistochemistry.

^a^ Guamanian ALS.

^b^ Study included one SOD1‐fALS patient.

Autopsy reports indicate widespread and early involvement of Bunina body pathology in ALS. In a patient who committed suicide only 5 months after diagnosis, Bunina bodies were already present in lower motor neurons [54]. In a case of frontal lobe dementia, possible motor involvement was only highlighted at autopsy via neurogenic atrophy of muscles of the tongue. Bunina bodies were already observable in the hypoglossal nuclei in this case, demonstrating their possible occurrence before motor symptoms present [55]. Indeed, FTD patients without clinical signs of ALS commonly demonstrate both TDP43 and Bunina bodies within lower motor neurons [52]. Conversely, a patient reliant on artificial ventilation support for the final 9 years of life demonstrated Bunina body pathology in rare anatomical locations such as the oculomotor nuclei and the medullary reticular formation [34]. This suggests that this pathology can be widespread and pervasively occur in rarely affected motor nuclei, as well as non‐motor areas, in long‐term illness.

Several proteins (e.g., p62, β‐amyloid precursor protein, α‐synuclein and tau) associated with neurodegeneration do not localise or associate with Bunina bodies [26, 56, 57, 58]. Unlike most other pathological inclusions, Bunina bodies are largely immuno‐negative for ubiquitin [26], although research is not always concordant and has sometimes demonstrated a small number of Bunina bodies to be ubiquitylated [46]. Whether this is due to cystatin C ubiquitylation or colocalisation of Bunina bodies to other ubiquitylated bodies is not known. TDP43 has been reported to occasionally colocalise with Bunina bodies, and TDP43 inclusions occur more frequently in neurons with Bunina bodies than in those without [59, 60]. The observation that TDP43 inclusions occur more frequently [28, 51], or even exclusively [30], in neurons containing Bunina bodies has been demonstrated via different techniques (immunofluorescent double labelling and sequential H&E staining and anti‐TDP43 immunohistochemistry) from different laboratories. The first study to examine this relationship showed that neurons containing TDP43 inclusions always exhibited Bunina bodies, whereas Bunina bodies were observed in neurons without TDP43 inclusions [30]. This reported only three patients, and an unspecified number of neurons, therefore limiting interpretation. Subsequently. Mori et al. performed serial H&E and TDP43 immunohistochemistry [51]. Bunina bodies were identified in 15 of 18 patients. Bunina bodies and TDP43 inclusions co‐occurred within lower motor neurons in 11 of these. Quantitatively, 33.9% of neurons with TDP43 inclusions contained Bunina bodies, and 81.8% of neurons with Bunina bodies also harboured TDP43 inclusions. The frequency of TDP43 inclusions was significantly higher in neurons with Bunina bodies than in those without [51]. This pathological association was later supported by a publication using largely the same cohort [28]. The significance of this colocalisation and juxtaposition remains unclear but suggests a relationship between the two inclusions and their formation processes. Underestimates in Bunina body pathology limit this type of research. Neurons that have lost free cytoplasmic cystatin C are much larger and thus less easily missed. Therefore, using this depletion of cytoplasmic cystatin C as a proxy for Bunina bodies may circumvent this problem: 98.9% of neurons with TDP43 inclusions have weak or no immunostaining for cytoplasmic cystatin C [27].

In 1993, Okamoto et al. [26] described Bunina bodies as being immunoreactive for cystatin C, which appears to be the primary aggregating component. Other proteins have since been associated with them: transferrin, peripherin, sortilin‐related receptor CNS expressed 2 and prosaposin also partially localise with Bunina bodies [53, 61, 62, 63]. However, they are best observed via immunohistochemistry with anti‐cystatin C antibodies. Although also observable via H&E staining, they are less distinct and therefore easily missed. Immunohistochemistry also captures the loss of free cytoplasmic cystatin C that accompanies Bunina body formation (Figure 1) and is therefore a more appropriate method. Furthermore, given their small size, Bunina bodies can be difficult to detect, even when they are present. These 1‐ to 5‐μm inclusions are found in motor neuron somata that can be as large as 50–100 μm in diameter. Therefore, unless an entire motor neuron is examined, it cannot be definitively claimed that no pathology is present within a neuron. This issue adds to the incompleteness of the current data, as it presents a difficulty in thoroughly detecting this pathology within the patient population. Multiple authors have highlighted that in cases where Bunina bodies are not initially found, they may be found when additional sections are examined [29, 36]. This is a major drawback of histological analysis of ALS pathology. Furthermore, Bunina bodies have once been reported in neuronal dendrites [64]; if this is a common phenotype, then pathology could be more widespread. Their prevalence within neuronal somas, processes, the central nervous system (CNS) and within the patient population is still in question because of variable sampling and lack of research. The only way to define aggregate burden and prevalence within the patient population is to conduct large‐scale neuropathological examinations via anti‐cystatin C immunohistochemistry in post‐mortem tissue. Tissue availability is a limiting factor in this.

## 3 Cystatin C

### 3.1 Structure and Functions

**FIGURE 3:**
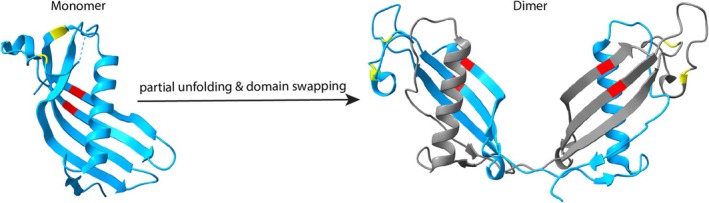
Crystal structure of monomeric and dimeric cystatin C formed by three‐dimensional domain swapping. Monomeric cystatin C (PDB 3GAX [65]) partially unfolds to dimerise (PDB 1G96 [66]) via three‐dimensional domain swapping. Cysteine residues highlighted in yellow (cys73 and cys83) and red (cys97 and cys117) form stabilising disulphide bonds [67]. Figure made in ChimeraX 1.10 [68].

Cystatin C, encoded by the *CST3* gene, is a 13.3‐kDa cysteine protease inhibitor within the cystatin type 2 superfamily. Like other type 2 cystatins, cystatin C consists of antiparallel beta sheets around a single alpha helix (Figure 3). It has two disulphide bonds at its carboxyl‐terminal, which are important for protein stabilisation [65, 69].

As a primarily secreted protein, cystatin C is synthesised as a preprotein, with an additional 26 amino acid signal peptide at its N‐terminus sequence, which mediates trafficking of the protein through the secretory pathway. This is cleaved to form the mature protein 120 amino acid protein. The active monomeric cystatin C protein functions primarily as an inhibitor of cysteine proteases, such as cathepsin B and S, by forming reversible, high‐affinity bonds with its targets. In the extracellular space, cystatin C plays a crucial role in regulating tissue remodelling and prevents excess extracellular matrix remodelling [70, 71, 72]. Other extracellular roles include immunomodulation and antimicrobial activities [73], inhibition of amyloid formation [74] and maintenance of the blood–brain barrier [75]. Cystatin C is also taken up from the extracellular environment, internalised by cells and targeted to lysosomes for protease inhibition [76, 77]. Although cystatin C is expressed by all nucleated cells and present in all bodily fluids [78], it is found in particularly high cytoplasmic concentrations within lower motor neurons [27]. This suggests a high cellular production or extracellular internalisation of cystatin C and therefore a specific role within these cells.

### 3.2 Aggregate Formation

In its physiological state, cystatin C is monomeric both in vitro and in vivo. However, several properties of its molecular structure, amino acid sequence, and possible modifications make it more prone to aggregation in the intracellular space.

Firstly, cystatin C can undergo three‐dimensional domain swapping, in which monomers exchange identical structural sub‐domains to form dimers or higher‐order oligomers (Figure 3) [66]. Domain swapping underpins cystatin C dimerisation, and cystatin C aggregation likely occurs via propagated domain swapping. This is a process in which long chains of associated monomers form, instead of the assembly of domain‐swapped cystatin C dimers [79]. Cystatin C domain swapping and fibril formation depend heavily on the local chemical environment—pH, temperature and protein concentration affect this propensity.

Secondly, mutations and protein modifications increase its amyloidogenicity. In Icelandic hereditary cystatin C amyloid angiopathy (HCCAA‐I), the most severe form of cerebral amyloid angiopathy (CAA), which affects the youngest patients, a dominant L68Q mutation in cystatin C increases the stable extracellular protein's rate of domain swapping. This readily produces aggregates and leads to amyloidosis and subsequent cerebral haemorrhage at an early age, with a mean age at death of approximately 30 years [80, 81]. Study of this L68Q mutation led to the identification of short peptide sequences within cystatin C's so‐called ‘aggregation‐prone core’, which further act as guides for fibril formation [69]. In these extracellular amyloid aggregates found within HCCAA‐I patients, N‐terminally truncated cystatin C, cleaved via elastase and lacking the first 10 amino acids [82], is the dominant form of L68Q mutant cystatin C. This truncated form has been shown to form dimers in the same domain‐swapped manner as full‐length cystatin C, albeit under different conditions and with a higher aggregation propensity [83].

Thirdly, cystatin C protein stability is linked to two disulphide bonds that cross‐link the three‐dimensional structure (see Figure 3) [84]. Disulphide bonds form through an oxidation reaction between two cysteine residues and are a common feature in secreted proteins. This process readily occurs in the oxidising extracellular environment to stabilise tertiary structures. Conversely, these bonds are destabilised by the reducing environment of the cellular cytoplasm, therefore making it more likely that the protein will aggregate even under relatively low intracellular concentrations. In support of this, dimerisation of the extremely stable chicken egg white cystatin (which shares significant structural and biological similarities to human cystatin C) has been shown to be accelerated by the breakage of its disulphide bonds [85]. Furthermore, destabilisation of cystatin C's disulphide bridges promotes domain swapping, whereas the addition of disulphide bonds provides additional stability, prevents dimerisation and reduces the transition to amyloid fibrils [65, 86].

Finally, phase separation is a phenomenon whereby a uniform mixture spontaneously divides into two or more liquid phases with differing component concentrations. This allows cellular membraneless compartments to form, which in turn permit complex biochemical reactions to occur. Recent focus has been placed on phase separation as a candidate mechanism for aberrant protein aggregation in neurodegeneration. Indeed, key proteins such as TDP43 have been shown to phase separate [87]. In addition, positively charged molecules are prone to phase separation within the cytoplasm due to molecular clustering in the presence of other large, negatively charged molecules (reviewed by Boeynaems et al. [88]). Cystatin C has a relatively high positive charge (isoelectric point [pI] = 9.2 [89]), which may increase its tendency to phase separate in the cytoplasm, and therefore promote its intracellular insolubility.

Clearly, the properties that promote cystatin C aggregation may be important in ALS. Indeed, cystatin C's ability to form higher molecular weight fibrils may underpin Bunina body formation. There are many questions that need to be answered surrounding cystatin C aggregation, specifically in the context of ALS. These include the following: (i) Are domain‐swapping oligomers or the N‐truncated form of cystatin C present in Bunina bodies? (ii) Does cystatin C undergo liquid–liquid phase separation? (iii) Could stabilisation of the protein's monomeric structure (as is successful in the treatment of transthyretin amyloidosis [90]) prevent Bunina body formation?

## 4 Cystatin C in Neurological Diseases

Cystatin C is highly expressed within the CNS. Indeed, its concentration is four times higher in cerebrospinal fluid (CSF) than in plasma [91]. Accordingly, cystatin C has been linked to many CNS disorders. As discussed above, the L68Q autosomal dominant mutation in *CST3* causes cystatin C amyloidosis, leading to haemorrhage, stroke and premature death in otherwise young individuals with HCCAA‐I [81, 92]. Furthermore, heterozygous truncating variants of *CST3* are linked to autosomal dominant adult‐onset leukodystrophy in eight reported families [93], and polymorphisms in *CST3* are linked to white matter lesions and cortical white matter changes leading to cognitive decline [94]. The A25T amino acid change, known as Variant B cystatin C, confers a change in the secretory targeting sequence of precursor cystatin C and conveys susceptibility to age‐related macular degeneration and Alzheimer's disease [95]. In the post‐mortem brain of Alzheimer's disease patients, cystatin C is colocalised with amyloid beta fibrils [96], and neuronal cystatin C is increased in the temporal lobe, most notably in vulnerable pyramidal neurons of cortical layers III and V [97]. In Parkinson's disease, increased serum levels of cystatin C correlate with cognitive decline and disease severity [98], and higher serum cystatin C levels also correlate with an increased risk of ischemic stroke [99].

## 5 Cystatin C in Human ALS

### 5.1 Cystatin C as a Biomarker for ALS

In ALS, aside from its aggregation into Bunina bodies within lower motor neurons, cystatin C levels are decreased in CSF. Its use as a biomarker has therefore been debated. In comparison to healthy controls or controls with other neurological diseases, cystatin C levels are most often found to be significantly decreased in CSF and increased in blood samples of ALS patients (Table 2). However, results are variable, possibly because of differing detection methods and small sample sizes. Whether extracellular cystatin C correlates with important disease parameters such as progression, ALS Functional Rating Scale–Revised (ALSFRS‐R) scores or survival time is debatable (see table for references). Furthermore, these extracellular measures may not accurately reflect intracellular pathological changes, as there are many other parameters influencing extracellular protein levels such as expression levels, solubility and excretion. At this stage, there does not appear to be a reliable relationship between CSF or blood cystatin C and diagnosis. However, it is possible that higher CSF cystatin C may predict longer survival [104].

**TABLE 2:** Summary of biomarker studies of cystatin C in ALS.

				Significance			
Ref.	Patients (m/f)	Sample	Method	ALS VS control groups	Progression	Duration	ALSFRS‐R
[100]	23 (?/?)	CSF ↓	SELDI‐TOF‐MS & WB	MCs SELDI‐TOF‐MS: *p* ≤ 0.01 MCs WB: *p* = 0.04	N/A	N/A	N/A
[101]	49 (33/16)	CSF ↓	SELDI‐TOF‐MS	HCs: *p* = 0.0005	N/A	N/A	N/A
[102]	14 (12/2)	CSF ↓	ELISA	DCs: *p* ≤ 0.024	N/A	NS	N/A
[103]	100 (67/33)	CSF ↓	SELDI‐TOF‐MS	HCs *p* = 0.002 DCs *p* = 0.01	N/A	NS	N/A
[104]	44 (31/13)	CSF ↓ Plasma ↑	ELISA	HCs CSF *p* = 0.038 HCs Plasma *p* = 0.001 DCs CSF—NS (*p* = 0.384) DCs Plasma—NS (*p* = 0.442)	N/A	CSF—NS Plasma—NS	N/A
[105]	31 (19/12)	CSF Serum	ITA	NS	N/A	N/A	N/A
[91]	92 (59/33)	CSF Serum	ITA	HCs CSF—NS HCs Serum—NS	CSF—NS Serum—NS	CSF—NS Serum—NS	CSF—NS Serum—*p* = 0.007 (neg)
[106]	20 (11/9)	CSF ↓	ELISA	DCs *p* < 0.05	N/A	NS	NS
[107]	356 (203/153)	Serum	ITA	N/A	*p* = 0.002 (pos)	N/A	*p* = 0.000084 (neg)
[108]	1086 (674/412)	Serum ↑	ITA	HCs—*p* < 0.001	*p* = 0.011 (pos)	N/A	*p* < 0.001 (neg)

Abbreviations: ↑, significantly increased; ↓, significantly decreased; CSF, cerebrospinal fluid; DCs, disease controls; ELISA, enzyme linked immunosorbent assay; HCs, healthy controls; ITA, immunoturbidimetric assay; MCs, mixed healthy and disease controls; N/A, not applicable; (neg), negative correlation; NS, non‐significant; (pos), positive correlation; SELDI‐TOF‐MS, surface‐enhanced laser desorption/ionisation–time of flight mass spectrometry; WB, western blot.

### 5.2 Studying *CST3* in ALS

No ALS‐causing mutations in the *CST3* gene have been found in a PCR screen of 57 sALS and 12 non‐SOD1 fALS [109]. However, genome‐wide association studies give a more comprehensive analysis of *CST3* in ALS. Using Genotype‐Tissue Expression (GTEx) [110] to identify expression quantitative trait loci (eQTLs) linked to *CST3* expression in CNS tissue, we have found a single‐nucleotide polymorphism is associated with elevated *CST3* expression (rs3004148, NES = 0.21, *p* = 1.2e‐7); the effect allele ‘T’ is associated with increased ALS risk (rs3004148, beta = +0.03, se = 0.01, *p* = 0.04388, REGENIE) [111]. This is consistent with a model whereby increased CNS expression of *CST3* leads to increased risk of ALS. Furthermore, as noted above, *CST3* mutations variously cause HCCAA‐I or a leukodystrophy. To our knowledge, such cases have not thus far been investigated for ALS‐relevant pathology.

### 5.3 Cystatin C in ALS Post‐Mortem Tissue

In neurologically healthy controls, cystatin C is observed in high cytoplasmic concentrations in lower motor neurons [27]. In comparison, in most sALS lower motor neurons, there is decreased cystatin C immunoreactivity: 81.1% of lower motor neurons have either weak or no anti‐cystatin C immunoreactivity compared to only 10.1% in neurologically healthy controls and 5.0% in controls with other neurological diseases, demonstrating disease specificity [27].

Mori et al. [27] showed that all neurons in sALS with an observable cystatin C‐positive Bunina body had this reduced free cytoplasmic cystatin C immunoreactivity, suggesting that Bunina bodies sequester the free protein. However, 75.1% of lower motor neurons without an observable Bunina body in the cell soma also had decreased cystatin C immunoreactivity. The observation of Bunina bodies in neural processes [64] may explain this: Bunina bodies may be present in a process that cannot be traced in the tissue section back to its respective neuronal soma to assess cytoplasmic cystatin C status. To our knowledge, dendritic Bunina bodies have only been documented once [64] but may be more widespread. Certainly, this is the authors' personal experience (see Figure 2H).

Varying reports exist regarding altered downstream effects of cystatin C pathology in ALS. An early immunohistochemistry report suggested that cathepsin B was specifically decreased in neurons containing Bunina bodies [112], although this has not been replicated [113]. cDNA microarray data from sALS spinal cord tissue demonstrate upregulated cathepsin B mRNA compared to neurologically normal controls [114]. The relevance of this is uncertain, as this reduction is not evident in mRNA from laser‐captured motor neurons [115]. RNA levels may not directly correlate with protein levels, and regulation of proteases by cystatin C may be at the protein, not mRNA, level. Definitive understanding of cystatin C downstream targets in ALS, such as cathepsins, would increase our knowledge of the potential pathological effects of soluble cystatin C reduction and Bunina body formation.

Changes in cystatin C have also been observed in non‐neuronal tissue. In ALS patient skin biopsies, epidermal cystatin C is significantly increased in comparison to healthy and disease controls [116], suggesting widespread alterations in cystatin C.

Although these reports can give valuable insight into the prevalence of pathology, the study of post‐mortem tissue is limited to end‐stage disease, and ALS models are needed to further understand neurodegenerative mechanisms at play.

## 6 Cystatin C in ALS Disease Models

Although human pathology demonstrates a clear correlation between Bunina bodies and disease, these methods cannot establish cause/effect relationships. Therefore, understanding the role of cystatin C in ALS can be supported by in vitro and in vivo models. Unfortunately, the data here are minimal and of little relevance to human disease. Firstly, cystatin C pathology is a feature seen only in cases with TDP43 pathology. Good animal models of TDP43 proteinopathy are few [117]. The use of SOD1 models is widespread yet may be particularly limiting for the study of cystatin C and Bunina body pathology as human *SOD1*‐fALS does not have Bunina body or TDP43 pathology [118]. Data from SOD1 models may yield generic insights into neurotrophism or toxicity but cannot usefully inform studies of MND/ALS with TDP43 pathology. Thus, cystatin C seems to be protective in SOD1‐G85R N2a models via inhibition of cathepsin B and mTOR‐mediated autophagy [119]. Intracerebroventricular administration of cystatin C at early‐stage disease in SOD1‐G93A mice increases survival time [110]. The finding of cystatin C aggregates within ventral horn motor neurons in these SOD1‐G93A mice [120, 121] is difficult to interpret especially as, in some cases, these differ in morphology from human Bunina bodies and are not accompanied by a loss of free cytoplasmic cystatin C [121]. Monomeric soluble cystatin C was significantly decreased, and Triton‐X‐100‐insoluble cystatin C oligomers with higher molecular weights (~50 kDa) were significantly increased in these cystatin C‐infused mice [120]. As these cystatin C‐infused mice had increased survival time, it may be that Bunina bodies have a neuroprotective function.

In TDP43‐based models of ALS, research into cystatin C remains limited. A C9orf72 poly‐PR primate model of ALS demonstrated that levels of cystatin C in CSF positively correlate with disease progression, as is seen in human disease [107, 108]. However, the authors did not investigate the presence of cystatin C aggregates in remaining motor neurons and reported no accumulation of TDP43 or phosphorylated TDP43 aggregates in brain tissue [122]. Direct evidence of cystatin C‐positive aggregates has been reported in a non‐human primate model overexpressing wild type TDP43 via adeno‐associated viral vector delivery into the cervical cord. Small cystatin C‐positive granules were observed in a subset of remaining lower motor neurons, notably only in those with exogenously expressed nuclear TDP43 [123]. Although these do somewhat resemble human Bunina bodies, a comparison to unaffected neurons has not been shown, therefore limiting interpretation. Notably, these granules do not colocalise with cytoplasmic TDP43. To our knowledge, this is the only model reporting cystatin C and TDP43 pathologies. No models of Bunina body pathology exist.

The paucity of relevant model system data exploring the relationship between TDP43 and cystatin C is a crucial issue to be addressed. Cystatin C perturbations in cell models of ALS have yet to be addressed. Certainly, sALS‐derived induced pluripotent stem cells and differentiated motor neurons are a logical and relevant starting point.

## 7 Cystatin C is Neuroprotective—Could Its Loss of Function Be a Factor in ALS Pathogenesis?

Bunina bodies are reported in 16.9% of lower motor neurons [28], yet the cytoplasmic cystatin C depletion that accompanies these inclusions is observed in 81% of lower motor neurons [27]. Given the prominence of the Bunina body in ALS, the role of free, soluble cystatin C is an important avenue to pursue further. It is likely that the reduction in free cytoplasmic cystatin C within ALS motor neurons leads to its loss of function. As these functions are neuroprotective and are probably diminished in disease, this could be a factor in driving pathogenesis. Indeed, in vitro and in vivo evidence suggests that cystatin C exerts these neuroprotective functions via multiple mechanisms, discussed below.

Firstly, cystatin C is an anti‐amyloidogenic protein: In its non‐aggregated form, it reduces the formation of amyloid beta fibrils in vitro and in vivo [74, 124, 125] and promotes the nonamyloidogenic processing of amyloid precursor protein (APP) in brain endothelial cells [126]. Overexpression of soluble cystatin C in an APP‐transgenic mouse model of Alzheimer's disease significantly reduced neocortical amyloid‐β fibril accumulation [127]. This amyloid clearance mechanism has been shown to act via microglial triggering receptor expressed on myeloid cells 2 (TREM2) activation, thereby promoting plaque degradation [128]. This is also seen in vitro when Aβ_1–40_ is incubated with monomeric cystatin C. Conversely, when incubated with aggregated cystatin C, the opposite is observed: Total amyloid‐β fibril levels were significantly increased, as shown by Thioflavin T fluorescence assay [129].

Secondly, cystatin C is a proautophagic protein. Autophagy is the cellular process responsible for the elimination of faulty proteins and damaged organelles and is key to homeostasis and maintaining neuronal health. This process is especially important in large post‐mitotic motor neurons, and there are clear links between autophagic health and neurodegeneration in several pathologies, including ALS [130]. Cystatin C has been reported to induce autophagy via inhibition of mTOR: Extracellular addition of cystatin C to N2a cells increases both the conversion of LC3‐I into LC3‐II, indicating formation of autophagosomes. These neuroprotective effects of cystatin C are prevented by inhibiting autophagy with 3‐methyladenine or *beclin 1* siRNA [131]. Furthermore, in in vivo Parkinson's models, application of cystatin C into the substantia nigra enhances neuronal autophagy and significantly decreases phosphorylated alpha‐synuclein [132]. Maintaining correct levels of autophagy is vital to neuronal health; a decrease in active monomeric cystatin C in motor neurons is likely detrimental.

Thirdly, the role of cystatin C as a cysteine protease inhibitor promotes neuronal survival. Knockdown of cathepsin B (a primary target of cystatin C) in mice slows motor neuron death after nerve injury, suggesting that cystatin C‐mediated inhibition of cathepsin B can also reduce neurotoxicity [133]. Furthermore, a rat model of intracerebral haemorrhage demonstrated increased cathepsin B expression. This elevated expression was reduced by cystatin C, which also alleviated severe brain oedema and neurological deficit scores, again demonstrating neuroprotective cystatin C functions [134].

Furthermore, in addition to the intracellular mechanisms described above, accumulating evidence suggests a neuroprotective role via its secretion. Extracellular vesicles loaded with cystatin C have been shown to be protective against cell death in primary neurons in vitro [135], and in a mouse model of stroke, extracellular vesicles administered intracerebroventricularly then demonstrate significantly increased synaptic markers, suggesting neuroprotection and synaptic preservation [136]. Mouse primary cortical neurons overexpressing Alzheimer‐associated presenilin 2 mutations had reduced cystatin C within secreted exosomes [137], suggesting that this reduction could be a contributing factor to disease. Together, these results demonstrate a neuroprotective role for cystatin C‐loaded extracellular vesicles. These protective functions suggest that therapeutic manipulation of cystatin C to elevate soluble, active protein levels could be a promising avenue for future interventions.

Finally, cystatin C is increased in response to oxidative stress. High‐oxygen conditions lead to oxidative stress‐induced cell death in cultured neurons, and cystatin C mRNA and protein levels are significantly upregulated in these neurons [138]. Further, cystatin C expression in PC12 neurons provides significant resistance to oxidative stress‐induced cell death [139], and in rat primary cortical neurons treated with hydrogen peroxide, the addition of cystatin C to culture media promotes cellular survival [131]. These data suggest that the increase in cystatin C may be protective.

To conclude, cystatin C exerts neuroprotective effects via inhibition of amyloid formation, promotion of autophagy, via cysteine protease inhibition and via its extracellular secretion. It is upregulated in response to cellular events such as oxidative stress, which is a known factor in ALS pathogenesis. Cystatin C levels are likely to be tightly regulated, and pathological cellular events that increase cystatin C mRNA and protein levels may disrupt the fine balance of its levels. This may make its aggregation more likely. It is possible that moderate increases in soluble cystatin C levels are neuroprotective, but in the reducing intracellular environment with the addition of extra disease‐associated events, the protein has a lowered solubility limit. Its aggregation into a Bunina body, as well as subsequent loss of function, is likely to confer a loss of neuroprotection. These aggregated species are then at best inactive or, at worst, pathogenic, which may contribute to disease pathogenesis.

## 8 Does Cystatin C Exacerbate Neurotoxicity?

Elevated cystatin C is reported in many neurodegenerative diseases, but whether this is the cause or result of the disease remains unknown. Therefore, although cystatin C has neurotrophic effects, it has been shown to be neurotoxic in some contexts, as discussed below.

Intrahippocampal administration of cystatin C in rats caused significant neuronal loss, which was negated by co‐administration of cathepsin B. This was also demonstrated in cystatin C‐treated cultured human neurons and was accompanied by an increase in DNA fragmentation and upregulation of caspase‐3, a key protein in the apoptotic pathway [140]. In vitro, dopaminergic neurons secreted cystatin C after toxin‐induced injury, which led to microglial activation and exacerbation of neuronal damage in a model of Parkinson's disease [141]. Further in vitro evidence comes from overexpression of cystatin C in neurons from differentiated PC12 cells, which then promoted tau phosphorylation and microtubule instability [142]. This mechanism could be acting through the unfolded protein response (UPR), a key signalling pathway that is upregulated by an overload of unfolded proteins and associated with tau phosphorylation during cellular stress [143]. This suggests a contribution of cystatin C to Alzheimer's disease pathogenesis and neurodegeneration.

The reasons why some authors report neuroprotection while others find toxicity when manipulating cystatin C levels remain unclear. It is evident that this manipulation impacts proteolysis, a very tightly regulated and sensitive cellular process that is impacted in ALS [130]. It is also clear that the correct ratio of cystatin C to its protease targets is crucial for neuronal health. There may be a very small concentration range of soluble cystatin C that is beneficial, and outside of this window, cystatin C aggregation is promoted, and dysregulated proteolysis occurs. Whether this neuroprotective window of soluble cystatin C is affected by cysteine protease availability or binding is unknown. Regardless, if these beneficial effects were to be harnessed in future, this window needs to be precisely defined. Finally, most data have looked at the application of soluble cystatin C. An interesting avenue of research for ALS would be to explore the potential toxic gain‐of‐function effects of Bunina body formation and how to mitigate this.

## 9 Interventions

Therapies that slow or prevent motor neuron degeneration in ALS are desperately needed. Targeting cystatin C to restore its neuroprotective functions or prevent its aggregation into Bunina bodies could provide an avenue for future interventions. Indeed, the natural reducing agent N‐acetyl‐cysteine (NAC) has already been shown to prevent the amyloid fibril formation of cystatin C in vitro [144] and to modulate disease‐related pathology in vivo in HCCAA‐I [81]. NAC has also been shown to inhibit cystatin C leakage from lysosomes in oxidative stress conditions and reduce its aggregation when mutant SOD1 is expressed in N2a cells [119]. Remarkably in HCCAA‐I patients, aggregation of the L68Q form of cystatin C is significantly reduced in skin biopsies and plasma of patients taking NAC supplements compared to disease controls [144, 145]. NAC acts as an antioxidant and increases levels of glutathione, which is depleted in ALS [146]. NAC has been trialled in ALS, demonstrating no statistically significant improvement in survival [147]. However, it has been argued that this initial trial was statistically underpowered and seemed to have opposing effects on different subtypes of disease, suggesting that further, larger trials are needed to fully assess this agent [148]. Furthermore, at present, it is unknown whether cystatin C undergoes fibril formation within Bunina bodies. Treatment with glutathione (another reducing agent) has already been tested in clinical trials and did not significantly improve outcomes [149, 150]. Unfortunately, glutathione does not readily cross the blood–brain barrier and is rapidly metabolised in blood [151]. Alternatives to oral glutathione administration have demonstrated promising results in other neurodegenerative diseases [152]. NAC‐amide or NAC ethyl ester, due to their more favourable pharmacokinetics [153, 154], could be trialled in ALS to target and reduce cystatin C aggregation in the hope that they act in a similar mechanism to that seen in HCCAA‐I, where they reduce cystatin C aggregation [145]. Notably, a clinical trial of NAC‐amide in HCCAA‐I patients is currently ongoing. Other avenues to target cystatin C, such as drug‐screen libraries and monoclonal antibodies, have shown the ability to inhibit cystatin C dimerisation as a treatment for HCCAA‐I [155]. These are exciting avenues for HCCAA‐I, in which the pathology is present in cerebral arteries. However, reducing cystatin C dimerisation in lower motor neurons in ALS has the added complexity of requiring these molecules to cross the blood–brain barrier and enter lower motor neurons. NAC‐amide and NAC ethyl ester possess pharmacokinetic properties that could enable them to achieve these goals.

## 10 Conclusions and Future Directions

To conclude, ALS, in common with other neurodegenerative diseases (e.g., Alzheimer's disease), is associated with at least two distinct proteinopathies—those involving cystatin C and TDP43. Although extensive research has been conducted on TDP43, the role of cystatin C and Bunina bodies in ALS remains poorly understood. The true prevalence of cystatin C depletion and Bunina bodies, both within neurons, neuronal processes and within the patient population, must be established to understand the extent of this pathology. To advance our understanding of cystatin C in ALS, in vivo models that better mimic ALS with TDP43 pathology must be used, as human *SOD1*‐fALS and therefore SOD1 models lack both TDP43 inclusions and Bunina bodies. Additionally, the relationship between these two pathological features of ALS warrants further study, as a deeper understanding of their interactions may give a more holistic understanding of the underlying pathomechanisms. Importantly, exploring the neurotrophic properties of cystatin C and how to preserve them could offer possible therapeutic strategies.

## Author Contributions

Conception of review: Sarah M. Granger and J. Robin Highley. Literature searching, draft of paper, writing and figure design: Sarah M. Granger; Scientific input and comments on draft: Sarah M. Granger, Rosemary A. Staniforth, Asbjorg Osk Snorradottir, Johnathan Cooper‐Knock, Kurt J. De Vos and J. Robin Highley.

## Funding

The authors would like to thank the Motor Neurone Disease Association for funding this work as a PhD studentship, which supports the first author.

## Conflicts of Interest

The authors declare no conflicts of interest.

## Data Availability

Data sharing not applicable to this article as no datasets were generated or analysed during the current study.
